# Different microbial and resistance patterns in primary total knee arthroplasty infections – a report on 283 patients from Lithuania and Sweden

**DOI:** 10.1186/s12891-021-04689-5

**Published:** 2021-09-17

**Authors:** Sujeesh Sebastian, Erdem Aras Sezgin, Justinas Stučinskas, Šarūnas Tarasevičius, Yang Liu, Deepak Bhushan Raina, Magnus Tägil, Lars Lidgren, Annette W-Dahl

**Affiliations:** 1 Faculty of Medicine, Department of Clinical Sciences Lund, Orthopedics, Lund University, Lund, Sweden; 2 Department of Orthopaedics & Traumatology, Aksaray University Training and Research Hospital, Aksaray, Turkey; 3 Department of Orthopedics & Traumatology, Lithuanian University of Health Sciences, Kaunas, Lithuania

**Keywords:** Drug resistance, Knee arthroplasty register, Microbial profile, Prosthetic joint infection, Polymicrobial infections

## Abstract

**Background:**

The microbiology and the susceptibility patterns of infected total knee arthroplasties (TKAs) vary depending on demographic, local antimicrobial stewardship, and surgical factors. We wanted to compare the recent microbial profile and antimicrobial resistance pattern in revisions due to infections after primary TKAs in Sweden and Lithuania. Our hypothesis was that there is a difference in bacteriology and resistance pattern based on patient related, societal and local hospital factors as almost similar praxis have been applied for TKA surgery, short term systemic prophylaxis and routine use of local gentamicin containing bone cement.

**Methods:**

Primary TKAs revised for the first time due to verified or suspected infection were collected nationwide in Sweden during 2018, and in Lithuania between 2011 and 2020 from a single major TKA revision centre in Kaunas. We identified 202 TKAs in Sweden from the Swedish Knee Arthroplasty Register and 84 from Kaunas revised due to infection. We collected available culture reports and evaluated the type of microorganisms with antimicrobial resistance pattern at revision.

**Results:**

The majority of the infected cases in Sweden were early-type prosthetic joint infection (PJI) (44%), whereas late-type PJI (52%) were more common in the Kaunas cases. Gram-positive bacteria prevailed in both Sweden (55%) and Lithuania (80%). *Staphylococcus aureus* was the most frequent organism identified in both countries (33% in Sweden and 34% in Lithuania). More polymicrobial infections were observed in Sweden than in Lithuania (16 and 6% respectively). Methicillin resistance in *Staphylococcus aureus* and coagulase-negative staphylococci were higher in Lithuania (4/28 and 19/29) than in Sweden (1/42 and 9/41).

**Conclusions:**

The type of infections, microbial profile, and drug resistance pattern differed between Sweden and Lithuania. Societal and local hospitals factors with emerging resistance in Lithuania are the most plausible explanation for the difference. Lack of complete data on a national level in Lithuania underlines the importance of adding microbiology of PJIs in implant registers for national aggregation and allow cross country comparisons.

**Supplementary Information:**

The online version contains supplementary material available at 10.1186/s12891-021-04689-5.

## Background

Prosthetic joint infection (PJI) is one of the most feared complications following total knee arthroplasty (TKA) and the most common reason for early failures. There are recent studies projecting alarming future rates of revision TKAs for PJI concomitant to the increase in primary TKAs [1]. Thus, in order to face the societal impact and challenge for the health care system, orthopedic surgeons, and infection specialists in cooperation need to be well prepared to prevent and manage PJIs.

Local microbiology and resistance patterns are key factors for effective prophylaxis and informed decision on surgical strategies. These may vary based on the general resistance pattern in the overall population in a geographical location, study population, type of systemic and local prophylaxis, implant, onset and severity of the PJI etc. [2]. Therefore, it is important to map and closely monitor the drug resistance evolution of pathogens responsible for PJIs, in order to tailor the prophylactic antibiotics and antimicrobial treatment policies.

There are several studies on microbial profiles of infected primary TKAs but the majority are limited by a small sample size, being single-center studies, microbial profile description based on type of PJI or type of surgical management [3, 4]. Though the previous three studies using Swedish Knee Arthroplasty Register (SKAR) data have shown the predominance of Gram-positive bacteria in knee PJIs, there are no recent reports on the microbial profile of primary TKA infections from Sweden [5–7]. The microbial profile of knee PJIs had not hitherto been reported by the Lithuanian Arthroplasty Register (LAR). We therefore evaluated the differences in microbial profile and resistance pattern of TKAs, revised due to infection, between Sweden and a single major TKA revision centre in Kaunas, Lithuania. We hypothesized there would be a difference in bacteriology and resistance patterns between two countries despite adopting almost similar perioperative praxis.

## Methods

Data were extracted from the SKAR, and LAR patients treated in Kaunas. Ethics approval covering the SKAR and the LAR data extraction was obtained by the ethics board of Lund University (LU20–02) and Lithuanian University of Health Sciences, Kaunas (No. 158200–16–832-371), respectively. The SKAR started in 1975 and the quality of the SKAR data has been validated and has a completeness of about 97% [8]. The LAR was established in 2011 and has a completeness of 90% but have not included microbiology data [9].

Using the SKAR, all patients who underwent primary TKA in Sweden between January 2010 and December 2018 were screened. Those patients revised due to suspected or verified infection for the first time between January 2018 and December 2018 were included in the study. When the same search strategy was applied in Lithuania where microbiological data of the infected TKA’s routinely not collected, limited data could only be obtained from Kaunas the largest revision center in Lithuania. This forced the authors to change the time period and patients who underwent primary TKA in Kaunas Klinikos between January 2011 and June 2020 were identified. Based on the anticipated large number of cases from SKAR for the same time period (2011–2020) and its probable large difference with LAR cases, we avoided the SKAR data evaluation for the mentioned time period. The Kaunas center is treating around 60% of all infected knee joint prosthesis in Lithuania. Re-revised cases, recurrent cases of infection, and infected cases of uni-compartmental knee arthroplasties in both countries were excluded from the study. For each patient, the following information were collected: age, sex, date of primary surgery, type of infection, type of surgical revision, type of pathogen, and its antibiotic susceptibility pattern.

In the SKAR, revision is defined as a “*new operation in a previously resurfaced knee in which one or more of the components are exchanged, removed or added (including arthrodesis or amputation)”* [8]. In the LAR, revision is defined as “*a second operation after an arthroplasty in which implant components are exchanged, removed or added*” [10].

Cloxacillin 2 g (3 doses) is the primary prophylactic antibiotic choice (> 92%) in Sweden [8]. For patients allergic to penicillin, cefotaxime 2 g (2 doses) or clindamycin 600 mg (2 doses) are used. In Kaunas, cefazolin 1 g (3 doses) for 24 h used as standard which from 2018 changed to cefazolin 2 g once. In both Sweden and Kaunas, more than 90% of the primary TKA’s uses vacuum-mixed bone cement containing gentamicin [8]. Optipac Refobacin and Palacos R + G Pro Prefilled are the predominant cement brands used in Sweden whereas Refobacin R, Palamed and BonOs R Genta bone cement predominates in Kaunas [8]. The most common type of implants used for primary TKAs in Sweden are NexGen MBT, PFC-MBT and Triathlon, and in Kaunas, NexGen LPS, Sigma PS and Scorpio PS are commonly used [8, 11].

PJI was defined as per the International Consensus Meeting 2018 (ICM 2018) criteria [12]. Using the Zimmerli classification of PJI with some modifications, PJIs were classified into 3 types based on the time of infection diagnosis after index surgery: early (< 3 months), delayed (between 3 months and 2 years), and late infections (> 2 years) [13].

Acute onset infections in a previously non-infected knee with documented or suspected previous bacteraemia were classified separately as acute haematogenous infections, irrespective of the time from arthroplasty. To be classified as an acute haematogenous infection there had to be a clear interval without signs of infection between the primary arthroplasty and the occurrence of infection [6]. Methicillin-resistant staphylococcal isolates were those showing non-susceptibility to one of the tested antibiotics (oxacillin or isoxazolyl-penicillins).

### Statistical analysis

Data are expressed as mean with range, or as numbers with percentage wherever applicable. Comparisons among groups (*Staphylococcus aureus*, coagulase-negative staphylococci (CoNS), and polymicrobial infections between Sweden and Lithuania), were performed using chi-square test or Fisher’s exact test in case of small numbers. A *p-*value of < 0.05 was considered statistically significant. All statistical analyses were performed with STATA version 15 (Stata Corp LLC, College Station, TX, USA).

## Results

**Fig. 1:**
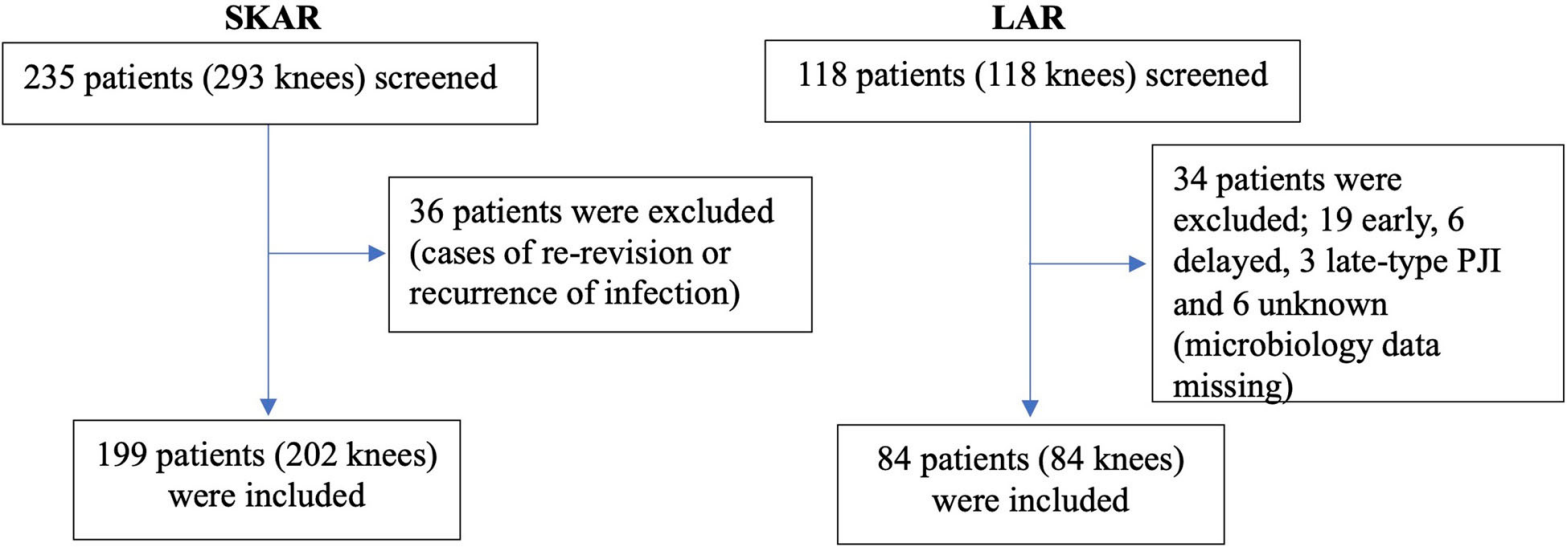
Flowchart of study population

From the SKAR, 235 patients (293 knees) were screened during the study period. Of the 235 patients revised due to infection, 36 were excluded due to being a re-revision case or recurrence of infection. One hundred ninety-nine patients (202 knees; three patients were bilaterally revised due to infection) were included (Fig. 1). During the study period, the LAR recorded 617 revised TKAs, whereof 243 were revised due to infection. Out of 243 infected TKAs, in Lithuania 118 were revised in Kaunas and of these 118 patients, only 84 patients had complete microbiological data (Fig. 1).

### Demographic and clinical data

61% of the infected cases from the SKAR were males, compared to 35% in LAR. The mean time interval between the TKA and PJI diagnosis was 17 months (range 0–132 months) for the cases from the SKAR and 43 months (range 0–218 months) for the cases from the LAR. The majority of the cases from the SKAR were early-type PJI (44%), whereas late-type PJI (52%) were the predominant type in the LAR (Table 1). Acute haematogenous type PJI cases were not reported from the LAR.

**Table 1:** Demographics, type of PJI and surgical revision in Sweden and Lithuania. DAIR = Debridement, antibiotics, irrigation and retention, ND = Not detected

Characteristic	SKAR (2018) *n* = 202 knees	LAR (2011–2020) *n* = 84 knees
Mean age (range)	69.8 (43–92)	70.2 (38–88)
Sex, n (%)		
Female	79 (39)	55 (65)
Male	123 (61)	29 (35)
Type of PJI, n (%)		
Early	88 (44)	11 (13)
Delayed	53 (26)	29 (35)
Late	39 (19)	44 (52)
Acute haematogenous	22 (11)	ND
Type of surgical revision, n (%)		
Two-stage revision	52 (26)	58 (69)
One-stage revision	0	8 (10)
Partial revision (DAIR)	150 (74)	18 (21)
Arthrodesis	0	0
Resection arthroplasty	0	0
Transfemoral amputation	0	0

### Microbial profile

Among the culture positive cases from Sweden, monomicrobial infections constituted 80% compared to 94% in Lithuania. Gram-positive organisms were more common in Lithuania than Sweden (80 and 55%, respectively), whereas Gram-negative PJIs were 11% in Lithuania and 4% in Sweden respectively (Table 2).

**Table 2:** Pathogens found in PJIs revised in Sweden and Lithuania

Pathogen	Sweden	Lithuania
	n (%)	n (%)
Aerobic Gram-positive	110 (55)	67 (80)
*Staphylococcus aureus*	42 (20.8)	27 (32)
CoNS	39 (19.3)	26 (30.9)
*Staphylococcus epidermidis*	23(11.4)	19 (23)
*Staphylococcus lugdunensis*	6 (3)	2 (2.3)
*Staphylococcus capitis*	6 (3)	1 (1.1)
*Staphylococcus caprae*	2 (1)	0
*Staphylococcus simulans*	1 (0.5)	0
*Staphylococcus hominis*	1 (0.5)	0
Other CoNS	0	4 (4.7)
*Staphylococcus* spp.	5 (2.5)	0
*Enterococcus faecalis*	5 (2.5)	2 (2.3)
Streptococci	19 (9.4)	12 (14.3)
*Streptococcus mitis*	3 (1.5)	0
*Streptococcus dysgalactiae Group G*	4 (2)	1 (1.1)
*Streptococcus agalactiae*	1 (0.5)	6 (7.1)
*Streptococcus pneumoniae*	2 (1)	0
*Streptococcus pyogens Group A*	1 (0.5)	4 (4.7)
β-Streptococcus group C-G	2 (1)	0
*Streptococcus sanguinis*	2 (1)	0
*Streptococcus anginosus (milleri group)*	1 (0.5)	1 (1.1)
*Streptococcus bovis*	2 (1)	0
Group C streptococci	1 (0.5)	0
Aerobic Gram-negative	8 (4)	9 (10.7)
*Pseudomonas aeruginosa*	1 (0.5)	0
*Escherichia coli*	2 (1)	1 (1.1)
*Serratia marcescens*	2 (1)	2 (2.3)
*Salmonella* Enteritidis	1 (0.5)	0
*Haemophilus parainfluenzae*	1 (0.5)	0
*Acinetobacter* spp.	1 (0.5)	1 (1.1)
*Acinetobacter johnsonii*	0	1 (1.1)
*Enterobacter cloacae*	0	2 (2.3)
*Enterobacter kobei*	0	1 (1.1)
*Proteus mirabilis*	0	1 (1.1)
Anaerobes	8 4)	0
*Cutibacterium acnes*	8 (4)	0
Other	2 (1)	3 (3.6)
*Cellulosimicrobium cellulans*	1 (0.5)	0
*Corynebacterium* spp.	1 (0.5)	1 (1.1)
*Bacillus circulans*	0	1 (1.1)
*Pasteurella multocida*	0	1 (1.1)
Polymicrobial	33 (16.3)	5 (5.9)
Negative	41 (20.3)	ND
Total	202	84

*CoNS* coagulase negative staphylococci, *ND* no data

Though staphylococcal infections were most common in both countries, they were more common in Lithuania than in Sweden (53/84, 63% and 86/202, 43%, respectively). In monomicrobial infections, *S. aureus* was the most frequent organism identified in both countries (42/128, 33% in Sweden and 27/79, 34% in Lithuania) followed by CoNS (39/128, 30% in Sweden and 26/79, 33% in Lithuania) (Table 2). We found no statistically significant difference in the distribution of *S. aureus* (*p*-value = 0.8) and CoNS (*p*-value = 0.7) between the countries.

### Type of PJI and microbial profile

According to the type of PJI, microbial profile differed from both the SKAR and Kaunas. In early PJI reported by the SKAR, polymicrobial infections were most common (24/88, 27.2%) followed by *S. aureus* (23/88, 26.1%) and CoNS (20/88, 22.7%) whereas in the Kaunas, *S. aureus* (8/11, 73%) was most common, followed by CoNS (3/11, 27%) (Table 3). In delayed and late PJI’s, culture-negative PJI’s predominated (15/53, 28.3% and 17/39, 43.5% respectively) in the SKAR but in Kaunas it was CoNS (9/29, 31% and 14/44, 31.8% respectively).

**Table 3:** The pathogens found in early, delayed, late and acute haematogenous infections in Sweden (2018) and Lithuania (2011-2020)

Type of Infection						
	Early, n (%)		Delayed, n (%)		Late, n (%)	
Pathogen	Sweden	Lithuania	Sweden	Lithuania	Sweden	Lithuania
*Staphylococcus aureus*	23 (26.1)	8 (73)	9 (16.9)	8 (27.5)	2 (5.1)	11 (25)
CoNS	20 (22.7)	3 (27)	11 (20.7)	9 (31)	6 (15.3)	14 (31.8)
*Staphylococcus* spp.	1 (1.1)	0	3 (5.6)	0	1 (2.5)	0
Streptococci	2 (2.2)	0	5 (9.4)	5 (17.2)	3 (7.6)	7 (15.9)
Enterococci	4 (4.5)	0	1 (1.8)	1 (3.4)	0	1 (2.2)
Gram-negative bacteria	3 (3.4)	0	3 (5.6)	2 (6.8)	1 (2.5)	7 (15.9)
Anaerobic bacteria	3 (3.4)	0	1 (1.8)	0	4 (10.2)	0
Other pathogens	0	0	1 (1.8)	1 (3.4)	1 (2.5)	2 (4.5)
Polymicrobial	24 (27.2)	0	4 (7.5)	3 (10.3)	4 (10.2)	2 (4.5)
Negative culture	8 (9)	ND	15 (28.3)	ND	17 (43.5)	ND
Total	88	11	53	29	39	44

*CoNS* Coagulase-negative staphylococci, *ND* No data

Acute haematogenous infections (*n* = 22) reported from Sweden: *Staphylococcus aureus*, 8 (36.3%); Coagulase-negative staphylococci, 2 (9%); Streptococci, 9 (40.9%); Gram-negative bacteria, 1 (4.5%); Polymicrobial, 1 (4.5%); Negative culture, 1 (4.5%). LAR did not report any acute haematogenous infections

### Polymicrobial infections

We found a statistically significant difference in the number of polymicrobial infections between Sweden and Lithuania, *n* = 33 (16.3%) and *n* = 5 (5.9%), respectively (*p*-value = 0.003). Of the 33 cases of polymicrobial infections in Sweden, 26 cases were infected with two pathogens, six cases with three and one case with four (Supplementary Table 1). Among the five cases of polymicrobial PJI reported from Kaunas, four were infected with 2 pathogens and the remaining one with three. Except three cases of *Staphylococcus epidermidis* + *Enterococcus faecalis* combination from SKAR data, no other predominance of any specific germ combinations was observed. The most common pathogen in polymicrobial infections from the SKAR was *S. aureus* (20.2%) followed by *S. epidermidis* (17.5%) and *E. faecalis* (12.1%). In Kaunas, the most common pathogen was *S. aureus* (18.1%) and *S. epidermidis* (18.1%) (Table 4).

**Table 4:** Distribution of pathogens in polymicrobial infections

Pathogen	SKAR	LAR
	*n* = 74	*n* = 11
Aerobic Gram-positive, n (%)		
*Staphylococcus aureus*	15 (20.2)	2 (18.1)
*Staphylococcus epidermidis*	13 (17.5)	2 (18.1)
*Staphylococcus lugdunensis*	3 (4)	1 (9)
*Staphylococcus capitis*	6 (8.1)	0
*Staphylococcus caprae*	2 (2.7)	0
*Staphylococcus hominis*	1 (1.3)	0
Other CoNS	0	1 (9)
*Enterococcus faecalis*	9 (12.1)	0
*Staphylococcus* spp.	2 (2.7)	0
*Streptococcus mitis*	1(1.3)	0
*Streptococcus agalactiae*	2 (2.7)	0
β-Streptococcus group C-G	1 (1.3)	0
Group B streptococci	1 (1.3)	0
Group A streptococci	1 (1.3)	0
*Streptococcus oralis*	0	1 (9)
Aerobic Gram-negative, n (%)		
*Pseudomonas aeruginosa*	3 (4)	0
*Escherichia coli*	1 (1.3)	0
*Serratia marcescens*	1 (1.3)	0
*Klebsiella pneumoniae*	2 (2.7)	0
*Enterobacter ludwigzi*	1 (1.3)	0
*Morgenella morgani*	1 (1.3)	0
*Enterobacter aerogenes*	2 (2.7)	0
*Citrobacter koserii*	1 (1.3)	0
*Proteus mirabilis*	0	1 (9)
Anaerobes, n (%)		
*Cutibacterium acnes*	1 (1.3)	0
*Finegoldia magna*	1 (1.3)	0
*Peptostreptococcus* spp.	0	1 (9)
Other*, n (%)*		
*Granulicatella adiacens*	3 (4)	1 (9)
*Moraxella osloensis*	0	1 (9)
Total	74	11

### Culture-negative infections

Sweden reported 20% cases as culture-negative whereas no data about culture-negative cases obtained from Lithuania (Table 2). In Sweden, culture-negative (CN) infections were most common in late-type infections (17/39, 44%) (Table 3).

### Organisms with antimicrobial resistance

The proportion of methicillin-resistant *Staphylococcus aureus* (MRSA) and methicillin-resistant coagulase-negative staphylococcus (MR-CoNS) was higher in Lithuania than Sweden (Table 5 and Supplementary Table 2 & 3). All the resistant strains of MR-CoNS from Sweden and Lithuania were strains of *S. epidermidis* except 1 in Lithuania. Among the staphylococcal isolates from Sweden and Lithuania, gentamicin resistance was highest in CoNS (13/44, 29.5% and 16/26, 61.5% respectively). No significant differences were found in antimicrobial resistance for Gram-negative organisms.

**Table 5:** Antibiotic susceptibility pattern of selected antibiotics from Sweden and Lithuania. Susceptibility to selected antimicrobial agents presented as the number of susceptible isolates divided by the number of all isolates tested against the respective agent and susceptibility percentages were given in brackets

	Gram-positive Aerobes								Gram-negative Aerobes			
Antimicrobial agents	*Staphylococcus aureus*		Coagulase-negative staphylococci		Streptococci		*Enterococcus faecalis*		Non-fermentative gram-negative aerobes		Other gram-negative aerobes	
	Sweden	Lithuania	Sweden	Lithuania	Sweden	Lithuania	Sweden	Lithuania	Sweden	Lithuania	Sweden	Lithuania
Methicillin*	41/42 (97.6)	24/28 (85.7)	32/41^a^ (78)	10/29^b^ (34.4)								
Gentamicin	46/47 (97.8)	20/25 (80)	31/44 (70.4)	10/26 (38.4)					3/3 (100)	1/2 (50)	5/5 (100)	5/7 (71.4)
Ciprofloxacin	47/48 (97.9)	23/26 (88.4)	45/59 (76.2)	19/27 (70.3)					5/5 (100)	1/2 (50)	14/15 (93.3)	5/5 (100)
T/S	45/45 (100)	29/29 (100)	45/58 (77.5)	22/29 (75.8)					1/2 (50)	1/1 (100)	14/14 (100)	2/2 (100)
Rifampicin	49/50 (98)	20/20 (100)	56/58 (96.5)	21/24 (87.5)								
Vancomycin	28/28 (100)	18/18 (100)	48/48 (100)	16/16 (100)	8/8 (100)	9/9 (100)	11/11 (100)					
Penicillin					19/20^c^ (95)	12/12 (100)						
Ampicillin							11/12 (91.6)	2/2 (100)				
P/T									4/4 (100)	1/2 (50)	14/14 (100)	6/6 (100)

* In Sweden and Lithuania, iso-penicillin and oxacillin respectively were used as surrogate antibiotics for methicillin susceptibility. T/S = Trimethoprim-sulfamethoxazole

P/T = Piperacillin- tazobactam. ^a^ = All resistant strains were *Staphylococcus epidermidis*. ^b^ = All resistant strains were *Staphylococcus epidermidis* except one. ^c^ = The penicillin-resistant isolate was a *Streptococcus pneumoniae*

## Discussion

Although infection is one of the most frequent complications in TKA, their microbial profile, resistance pattern, and how they differ between different countries, are however still grey areas that need to be explored further to frame local and/or national policies. In this study, microbiological data from two geographically close northern countries but with different initial start and evolution of TKA were compared and, differences were found in the antimicrobial susceptibility pattern and type of infections.

There are several limitations in this study. The SKAR microbiology data was extracted for 1 year 2018, but LAR data were collected for a longer period, since 2011. Although, we aimed for a comparable time period, with current technical lacunae in LAR microbiological data collection, only few cases were obtained from LAR for 2018 and extending the time points was unavoidable. Based on the anticipated large number of cases from SKAR for the same time period (2011–2020), which could lead to much more included cases (15–20 times) in Sweden compared to Lithuania, we avoided the SKAR data evaluation for the mentioned time period. With a data completeness of over 95% and the large cohort of infected cases, we however believe that the SKAR data is representative of the recent trends from Sweden. Secondly, data from Sweden were based on a national data and from Lithuania collected from one major center where according to LAR data two thirds of all the primary infected TKAs in the country were revised. The reason for this could be attributable to the current data collection system of LAR register and point towards the importance of linking microbiological data with national registers. We hope dissemination of these limitations ultimately could lead to the improvement of existing data collection systems. Lastly, complete data on acute haematogenous type PJI and CN-PJI were not reported in Lithuania. The lack of evidence-based treatment policies for knee PJIs in Lithuania recently reported may have affected the quality of the data as well [10].

The differences in baseline demographic and clinical data from both countries should also be considered while interpreting the results. Along with the observed gender difference in two patient populations, the type of PJI varied between the two countries, which in turn was reflected in the choice of surgical revision. A multitude of patient related and surgical factors which are known for its relation to revision and infection following primary arthroplasties such as primary joint disease, co-morbidities, antimicrobial prophylaxis, type and brand of bone cement used, antibiotic bone cement mixing technique, patellar resurfacing, types of polyethylene, and implant model/brand might have also affected our results [8, 11]. In addition, the general resistance pattern, large difference in the size of the general population, health care system and socioeconomic background of the patients could to some extent have contributed to observed differences. There are local hospital factors which also need to be considered such as type of hospital (university/county/private hospital), surgical routine and experience of the individual surgeons, and their degree of adherence to the standard treatment protocols just to mention a few. While Sweden was early in adopting total joint arthroplasty, it has a relatively short history in Lithuania [14]. Also, as previously reported the antibiotic prescription strategies specifically in patients following primary TKA in Lithuania could be another local factor that influenced the results [10].

In congruence to previous register-based and multicenter reports, Gram-positive aerobic PJI dominated both in Sweden and Lithuania [6, 15–17]. Although data were obtained from single centers, recently Villa et al. reported a similar trend from 6 countries covering 3 continents [18]. Few studies reported contradictory findings with approximately two-thirds of Gram-negative PJIs (GN-PJIs) [4, 19]. All these studies were conducted at single speciality centers and the majority of the cases were referred cases from other hospitals.

*S. aureus* was the most common pathogen responsible for infections in both Sweden and Lithuania followed by CoNS. In two separate studies, using more than 350 infected knee arthroplasties from the SKAR, Bengtson et al. and Stefánsdóttir et al. reported similar trends [5, 6]. The majority of the previous large studies on PJIs of TKA also noted the high frequency of *S. aureus* and CoNS [16, 17, 20]. This in turn highlights that, irrespective of the changes in proportions of the individual pathogen types, staphylococcal infections, precisely *S. aureus* and CoNS continues to be the most common pathogens responsible for PJIs.

In our study, almost half of the reported PJIs from Sweden were of early-type and this is in agreement with previous SKAR reports [5, 6] while in Lithuania, late-type PJI dominated. Due to heterogeneity in the PJI classification schemes used, studies aimed at a particular type of PJI, or reporting data by combining different prosthetic joints, make it difficult to make comparisons on trends based on the literature [15, 21, 22]. However, limited current data indicate variation in type of PJI between different countries or geographical locations.

Polymicrobial infections were common in early infections in the SKAR, as reported earlier [6]. The preponderance of *S. aureus* in both countries corroborates the role of this virulent bacterial strain in early-type infections. We also found higher number of patients with CoNS infections in early PJI. Contrary to the high prevalence of Gram-negative aerobes in early-type infections reported in previous studies [17, 23], the prevalence we observed was low. This could be attributable to the overall low incidence in our study, which has been reported earlier by Swedish studies [6]. CoNS is known to be one of the most common bacteria in delayed- or late-onset PJIs [21]. In current study, their distribution varied between two countries especially in late onset infections. This could be due to the lack of reporting of CN-PJI and lots of missing data from Lithuania. Along with these problems, as well as the diagnostic challenges associated with CoNS, probably there might be an underestimation of CoNS PJI which needs to be explored in future studies.

Although the rate and microbiology of polymicrobial infections from both countries were in agreement with previous reports [5, 24], there was a significant difference between the two countries and this could be explained by the more early type PJI reported in Sweden, where predominance of polymicrobial infections are well known. These findings are important since patients with polymicrobial infections were reported to have a higher failure rate, with amputation, arthrodesis and infection-related mortality compared to patients with monomicrobial infections [25]. In addition, there is a lack of treatment protocols for polymicrobial infections in current guidelines such as the Infectious Disease Society of America guidelines on PJI [26]. Owing to the coverage for both Gram-positive and Gram-negative bacteria, Tan et al. suggested the combination of cefazolin and gentamicin for polymicrobial infections [25]. Interestingly, in Lithuania systemic cefazolin and local gentamicin were used during primary TKAs. This factor along with low prevalence of early infections in Lithuania may have a role in the observed difference in polymicrobial infections between the two countries.

In Sweden, the frequency of culture-negative infections was high (20%) and is a matter of concern as they are associated with poor outcomes and a higher rate of salvage procedures [27]. The majority of the CN-PJIs in our study, were late and delayed infections, in which predominance of CN-PJIs are well known [28]. Previous or ongoing antibiotic treatment, culture techniques and incubation time used could have affected the culture results. However, current study lacks data on these possible factors for CN-PJI. Further improvements in the diagnosis protocols for the accurate identification of the responsible pathogens in PJIs are warranted with special attention to the use of biofilm detachment techniques in late and delayed type infections.

As observed in our study, the Nordic countries are known for a low MRSA prevalence [6, 16]. Strict antibiotic societal usage policies could be one reason. Though the prevalence of MRSA was higher in Lithuania than in Sweden, it was still in line with what is reported from other countries [20, 29]. Other factors that need to be taken into account are the widespread use of antibiotics in livestock and overall increase in antibiotic consumption contributing to emerging resistance in the general population [30]. The use in husbandry might be higher in Lithuania compared to Sweden.

The changing virulence pattern of CoNS, especially of *S. epidermidis* is well documented [17] and we noted high resistance shown by isolates of *S. epidermidis*. However, compared to previous reports from Sweden, our study actually showed less resistance in CoNS isolates [6]. This needs to be followed in additional longitudinal studies.

Studies indicate that antibiotic-loaded bone cements (ALBCs) reduce the risk of infections following primary knee arthroplasties, but the risk of developing drug-resistant strains is one of the reasons against its routine use [31, 32]. The resistance pattern of *S. aureus* and CoNS isolates to gentamicin varied between Sweden and Lithuania. But, although bone cement with gentamicin used in majority of the cemented primary TKA’s in both countries, the lack of detailed data prevents us from drawing any conclusions on the observed differences. However, we believe that the current findings imply the multifactorial aspects in the evolution of drug resistance in PJI rather than local ALBC use as the potential cause.

## Conclusions

The most common bacteria causing knee PJIs were similar in Sweden and Lithuania but with different antimicrobial susceptibility profile and type of infections. Various factors such as patient related, societal and local hospital factors might be related to the observed differences but needs to be confirmed in future longer studies with larger comparable cohorts. With the current limitations of registers and descriptive nature of the study, a detailed evaluation of the underlying causes and warranting the change of current clinical practises is not possible. The role of aggregated reporting of microbiological data included in national registers is recommended.

## Supplementary Information

**Additional file 1: Supplementary Table 1.** Distribution of bacterial combinations in polymicrobial infections. **Supplementary Table 2.** Number of pathogens tested for and susceptibility to selected antimicrobial agents in patients with PJI reported from Sweden. Susceptibility to selected antimicrobial agents presented as the number of susceptible isolates divided by the number of all isolates tested against the respective agent. **Supplementary Table 3.** Number of pathogens tested for and susceptibility to selected antimicrobial agents in patients with PJI reported from Lithuania. Susceptibility to selected antimicrobial agents presented as the number of susceptible isolates divided by the number of all isolates tested against the respective agent.

## Data Availability

The datasets used and/or analysed during the current study are available from the corresponding author on reasonable request.

## Abbreviations

ALBCs: Antibiotic-loaded bone cements
CoNS: coagulase-negative staphylococci
CN-PJI: Culture negative prosthetic joint infections
LAR: Lithuanian Arthroplasty Register
MRSA: Methicillin-resistant *Staphylococcus aureus*
MR-CoNS: Methicillin-resistant coagulase-negative staphylococcus
PJI: Prosthetic joint infection
SKAR: Swedish Knee Arthroplasty Register
TKA: Total knee arthroplasty
