# Supplementary material for: Different microbial and resistance patterns in primary total knee arthroplasty infections – a report on 283 patients from Lithuania and Sweden
Source: BMC Musculoskelet Disord. 2021 Sep 17;22:800. doi: 10.1186/s12891-021-04689-5 (PMC8449428; doi:10.1186/s12891-021-04689-5)
Supplement: Supplementary file 1 — Additional file 1: Supplementary Table 1. Distribution of bacterial combinations in polymicrobial infections. Supplementary Table 2. Number of pathogens tested for and susceptibility to selected antimicrobial agents in patients with PJI reported from Sweden. Susceptibility to selected antimicrobial agents presented as the number of susceptible isolates divided by the number of all isolates tested against the respective agent. Supplementary Table 3. Number of pathogens tested for and susceptibility to selected antimicrobial agents in patients with PJI reported from Lithuania. Susceptibility to selected antimicrobial agents presented as the number of susceptible isolates divided by the number of all isolates tested against the respective agent. [file 12891_2021_4689_MOESM1_ESM.docx]

**Supplementary Table 1:** Distribution of bacterial combinations in polymicrobial infections

| Sweden | | Lithuania | |
| --- | --- | --- | --- |
| Patient Number | **Bacterial combinations** | **Patient Number** | **Bacterial combinations** |
| 4 | *Staphylococcus hominis + Pseudomonas aeruginosa* | 20 | *Streptococcus oralis +* Granulicatella adiacens + *Moraxella osloensis* |
| 5 | *Staphylococcus epidermidis +* Group B streptococci | 42 | *Staphylococcus lugdunensis + Proteus mirabilis* |
| 7 | *Staphylococcus epidermidis + Staphylococcus lugdunensis* | 56 | *Staphylococcus aureus + Staphylococcus epidermidis* |
| 15 | *Staphylococcus epidermidis + Enterococcus faecalis* | 67 | *Staphylococcus aureus +* other CoNS |
| 21 | *Staphylococcus* spp*. + Streptococcus mitis* | 71 | *Staphylococcus epidermidis + Peptostreptococcus* spp*.* |
| 22 | *Staphylococcus aureus + Enterococcus faecalis + Enterobacter ludwigzi* |  |  |
| 29 | *Staphylococcus aureus + Klebsiella pneumoniae* |  |  |
| 37 | *Staphylococcus aureus +* Group A streptococci |  |  |
| 39 | *Staphylococcus epidermidis + Staphylococcus capitis* |  |  |
| 41 | *Staphylococcus aureus + Cutibacterium acnes* |  |  |
| 48 | *Staphylococcus capitis + Staphylococcus caprae* |  |  |
| 51 | *Staphylococcus aureus + Staphylococcus epidermidis + Streptococcus agalactiae* |  |  |
| 63 | *Staphylococcus aureus + Staphylococcus epidermidis + Enterococcus faecalis* |  |  |
| 68 | *Staphylococcus capitis + Staphylococcus lugdunensis* |  |  |
| 76 | *Staphylococcus aureus + Staphylococcus spp* |  |  |
| 84 | *Staphylococcus epidermidis + Enterococcus faecalis* |  |  |
| 89 | *Staphylococcus aureus + Enterococcus faecalis + S. marcescens* |  |  |
| 92 | *Staphylococcus aureus + Pseudomonas aeruginosa* |  |  |
| 98 | *Staphylococcus epidermidis + Morgenella morgani* |  |  |
| 99 | *Staphylococcus aureus +* β-Streptococcus group C-G |  |  |
| 100 | *Enterococcus faecalis + Enterobacter aerogenes + Pseudomonas aeruginosa + Citrobacter koserii* |  |  |
| 102 | *Staphylococcus aureus + Enterococcus faecalis* |  |  |
| 112 | *Staphylococcus capitis + Enterococcus faecalis* |  |  |
| 118 | *Escherichia coli + Klebsiella pneumoniae* |  |  |
| 130 | *Staphylococcus aureus + Enterobacter aerogenes* |  |  |
| 134 | *Staphylococcus epidermidis + Enterococcus faecalis* |  |  |
| 148 | *Staphylococcus epidermidis + Staphylococcus capitis +* Granulicatella adiacens |  |  |
| 161 | *Staphylococcus aureus +* Granulicatella adiacens |  |  |
| 163 | *Streptococcus agalactiae +* Granulicatella adiacens |  |  |
| 170 | *Staphylococcus aureus + Staphylococcus epidermidis + F. magna* |  |  |
| 177 | *Staphylococcus aureus + Staphylococcus lugdunensis* |  |  |
| 199 | *Staphylococcus epidermidis + Staphylococcus caprae* |  |  |
| 203 | *Staphylococcus epidermidis + Staphylococcus capitis* |  |  |

**Supplementary Table 2**: Number of pathogens tested for and susceptibility to selected antimicrobial agents in patients with PJI reported from Sweden. Susceptibility to selected antimicrobial agents presented as the number of susceptible isolates divided by the number of all isolates tested against the respective agent.

| Antimicrobial agents | | | | | | | | | | | | | | | | | |
| --- | --- | --- | --- | --- | --- | --- | --- | --- | --- | --- | --- | --- | --- | --- | --- | --- | --- |
| **Pathogens** | **PEN** | **AMP** | **Iso Pen** | **CFX** | **TAX** | **CAZ** | **PIP-TZB** | **IMI** | **GM** | **TOB** | **ERY** | **CLI** | **FUS** | **TMP-SMX** | **CIP** | **VAN** | **RIF** |
| **Gram-positive Aerobes**  *Staphylococcus aureus*  Coagulase-negative staphylococci  *Staphylococcus* spp*.*  Streptococci  *Enterococcus faecalis* | 19/20^c^ | 11/11  11/12 | 41/42^a^  32/41^b^  5/5 |  | 16/16 |  | 8/8 | 5/5  7/7 | 46/47  31/44  5/6  1/2  1/3 | 26/27  30/36  2/3  1/1  0/1 | 1/1  7/8  1/2  14/15 | 56/57  47/62  6/7  21/24  1/3 | 55/56  42/58  5/7 | 45/45  45/58  4/4  10/13 | 47/48  45/59  6/7  3/4  0/2 | 28/28  48/48  4/4  8/8  11/11 | 49/50  56/58  6/6 |
| **Gram-negative Aerobes**  *Pseudomonas aeruginosa*  *Escherichia coli*  Serratia marcescens  *Klebsiella pneumoniae*  *Enterobacter aerogenes*  *Enterobacter ludwigzi*  *Morgenella morgani*  *Citrobacter koserii*  Salmonella Enteritidis  Haemophilus parainfluenzae  *Acinetobacter* spp. |  | 1/1 |  | 1/1  1/1 | 0/2  3/3  3/3  2/2  1/1  1/1  1/1  1/1  1/1 | 3/3  1/1  3/3  2/2  1/1  1/1  1/1  1/1  1/1 | 4/4  3/3  3/3  2/2  2/2  1/1  1/1  1/1  1/1 | 1/1  1/1  0/1  1/1  1/1 | 2/2  1/1  2/2  1/1  1/1  1/1 | 2/2  2/2  2/2  2/2  1/1  1/1  1/1 |  |  |  | 0/1  3/3  3/3  2/2  2/2  1/1  1/1  1/1  1/1  1/1 | 4/4  3/3  2/3  2/2  2/2  1/1  1/1  1/1  1/1  1/1  1/1 | 1/1 |  |
| **Anaerobes**  *Propionibacterium acnes*  *Finegoldia magna* | 8/8  1/1 |  |  |  |  |  | 6/6  1/1 | 3/3 |  |  |  | 9/9  1/1 |  |  |  |  |  |

PEN, penicillin; AMP, ampicillin; CFX, cefuroxime; TAX, cefotaxime; CAZ, ceftazidime; PIP-TZB, piperacillin-tazobactam; IMI, imipenem; GM, gentamicin; TOB, tobramycin; ERY, erythromycin; CLI, clindamycin; FUS, fusidic acid; TMP_SMX, trimethoprim sulfamethoxazole; CIP, ciprofloxacin; VAN, vancomycin; RIF, rifampicin.

^a^The isolate not susceptible to the agent in question was defined as methicillin-resistant *S. aureus* (MRSA).

^b^The isolate not susceptible to the agent in question was defined as methicillin-resistant coagulase-negative staphylococci (MR-CoNS). All 9 resistant strains were *Staphylococcus epidermidis*. ^c^The penicillin-resistant isolate was a *Streptococcus pneumoniae*.

**Supplementary Table 3**: Number of pathogens tested for and susceptibility to selected antimicrobial agents in patients with PJI reported from Lithuania. Susceptibility to selected antimicrobial agents presented as the number of susceptible isolates divided by the number of all isolates tested against the respective agent.

| Antimicrobial agents | | | | | | | | | | | | | | | | | | | |
| --- | --- | --- | --- | --- | --- | --- | --- | --- | --- | --- | --- | --- | --- | --- | --- | --- | --- | --- | --- |
| **Pathogens** | **PEN** | **AMP** | **OXA** | **CFX** | **TAX** | **CAZ** | **PIP** | **PIP-TZB** | **IMI** | **GM** | **TOB** | **AMP-SUL** | **ERY** | **CLI** | **TET** | **TMP-SMX** | **CIP** | **VAN** | **RIF** |
| **Gram-positive Aerobes**  *Staphylococcus aureus*  Coagulase-negative staphylococci  Streptococci  *Enterococcus faecalis* | 12/12 | 2/2 | 24/28^a^  10/29^b^ |  | 7/8 |  | 1/1 |  |  | 20/25  10/26 |  |  | 20/20  6/15  8/10 | 22/22  18/24  8/9 | 16/19  10/14 | 29/29  22/29 | 23/26  19/27 | 18/18  16/16  9/9 | 20/20  21/24 |
| **Gram-negative Aerobes**  *Escherichia coli*  Serratia marcescens  *Acinetobacter* spp.  *Enterobacter* spp.  *Proteus mirabilis* |  | 0/1  0/1  0/1 |  | 1/1  2/3  1/2 | 3/3  1/2 | 1/1  1/1 | 0/1  0/1  1/1 | 1/1  1/2  3/3  2/2 | 2/2  1/1 | 1/1  1/1  1/2  3/3  0/2 | 1/1 | 0/1  1/3  1/2 |  |  | 1/1 | 2/2  1/1 | 1/2  3/3  2/2 |  |  |
| **Anaerobes**  *Peptostreptococcus* spp. | 1/1 |  |  |  |  |  |  |  |  |  |  |  |  |  | 1/1 |  |  |  |  |

OXA, oxacillin; PIP, piperacillin; AMP-SUL, ampicillin-sulbactam; TET, tetracycline.

^a^The isolate not susceptible to the agent in question was defined as methicillin-resistant *S. aureus* (MRSA).

^b^The isolate not susceptible to the agent in question was defined as methicillin-resistant coagulase-negative staphylococci (MR-CoNS). All resistant strains were *Staphylococcus epidermidis* except one.
